# Means of intracellular communication: touching, kissing, fusing

**DOI:** 10.15698/mic2021.05.747

**Published:** 2021-04-13

**Authors:** Anne Spang

**Affiliations:** 1 Biozentrum, University of Basel, Klingelbergstrasse 70, CH-4056 Basel, Switzerland.

**Keywords:** intracellular traffic, membrane contact sites, kiss-and-run, membrane fusion, signalling

## Abstract

Eukaryotic cells are complicated factories that need ensure productivity and functionality on the cellular level as well as being able to communicate with their environment. In order to do so cells developed intracellular communication systems. For a long time, research focused mainly on the secretory/biosynthetic and endocytic routes for communication, leaving the communication with other organelles apart. In the last decade, this view has changed dramatically and a more holistic view of intracellular communication is emerging. We are still at the tip of the iceberg, but a common theme of touching, kissing, fusing is emerging as general principles of communication.

## INTRODUCTION

Eukaryotic cells contain a variety of organelles that need to communicate with each other for cellular function. The perhaps best studied communication system is the secretory pathway in which cargo is transferred from the endoplasmic reticulum (ER) to the Golgi apparatus in transport carriers, so-called vesicles. In the Golgi, these cargoes are modified and are transported within the cisternae as they mature from cis to trans. This again, involves transport vesicles that retrieve glycosylation enzymes and other proteins, that are resident of the previous cisterna, comparable to the distillation process, in which everything is removed which should not be at this stage anymore. Finally, at the trans Golgi, cargo is segregated into different parts of a network - the trans-Golgi network (TGN) - which generates different transport vesicles that go to various locations such as the plasma membrane, different endosomes, or the lysosome. The counterpart of the secretory pathway, the endocytic pathway operates under similar premises.

In recent years it became clear that communication between organelles is much more elaborate. Communication cannot only be restricted to organelles along the secretory pathway but must also include other organelles such as mitochondria, lipid droplets (LD) and peroxisomes. However, vesicular transport between organelles outside the biosynthetic and endocytic routes had not been observed. In contrast, it was known for a long time that organelles would touch each other in the cell. These encounters were widely thought to be due to space restrictions and crowdedness. This view has drastically changed during the last decade.

## TOUCHING

The discovery of membrane contact sites as means of communication between organelles as well as organelles and the plasma membrane has changed the concept of intracellular communication [1–4]. At membrane contact sites, two organelles touch each other and through the contact site, lipids, ions, and potentially small molecules can be transferred from one organelle to the other. For example, phosphatidylserine (PS) is transferred from the ER to mitochondria, where it becomes decarboxylated to generate phosphatidylethanolamine (PE), which is then incorporated into various organellar membranes. Likewise, cholesterol is transferred from endolysosomes to the ER, and fatty acids flow from LDs to mitochondria for β-oxidation in mammalian cells [5, 6]. Again, between the ER and mitochondria, Ca^2+^ is exchanged with the help of VDAC and IP3R [7]. These entities are dynamic and can change probably in number and size depending on the cellular state. Of note, the ER makes contact with all membrane-bound organelles, which might also explain its important role in stress response [8]. Thus, all membrane bound organelles can communicate with each other, and there is an ever-growing number of protein factors present at contact sites and some are important for the formation of these sites. Contact sites can be long-lived or highly dynamic, also the surface of the contact is highly variable. Besides these contacts where membranes touch, there are other contact sites, in which organelles can be 30 nm apart, like in mitochondria associated membranes (MAMs), clearly a distance too big to transfer lipids and ions. It was proposed that they act as signalling hubs, but the underlying mechanisms remain enigmatic [9]. Moreover, there is still very scarce knowledge about the regulation of the different contact sites. It is believed that the distance of plasma membrane – ER contacts could be regulated by the Ca^2+^-bound state of the extended synaptotagmins/tricalbins, which are structural components of these contact sites [10–13]. However, whether this is a more general mechanism to regulate contact sites remains to be seen. Membrane remodellers and regulators such as small GTPases, in particular of the Arf/Sar family, appear to play a critical role [14, 15], but there is still a significant gap of knowledge to fill before we really understand this type of intracellular communication.

## KISSING

Kissing or better kiss-and-run was initially discovered as an intercellular communication system between neurons. Synaptic vesicles would be tethered at the plasma membrane and upon stimulation, fusion of the tethered vesicle with the plasma membrane would be triggered and neurotransmitter would be released. This fusion, however, would not result into membrane flattening, but rather the vesicular shape would be maintained. The synaptic vesicle could either stay tethered and poised for the next quantal release of neurotransmitter or go off and come back. This process was then coined kiss-and-run. More recently kiss-and-run has also been observed on intracellular membranes. For example, Rab11 recycling vesicles kissing sorting endosomes to take up cargo, such as transferrin receptor and the glucose transporter GLUT1, to be brought back to the plasma membrane [16]. In addition, kiss-and-run has been proposed between endosomes and lysosomes or for the transfer of iron between endosomes and mitochondria [17]. In the case of the recycling-sorting endosome interaction the tether and parts of the fusion machinery have been identified, while this remains enigmatic in other cases. In comparison to membrane contact sites, the two entities involved undergo membrane fusion and fission, and in the fused state, they can exchange material without losing their identity. The material comprises also transmembrane and soluble, lumenal proteins, which are not exchanged at membrane contact sites. It remains unclear to date how widespread kiss-and-run as communication method is between organelles, but we have only started to scratch at the surface. The detection of these events was made possible with the technological advance in fast, high resolution imaging techniques, as the kisses usually only last seconds [16]. Thus, in the future we should be in a better position to record and to interrogate these types of events and understand their regulation. In fact, kiss-and-run of vesicles in Golgi to promote bi-directional cargo transport between Golgi cisternae had been proposed in the past [18], and the idea gained more traction more recently [19, 20]. Moreover, the discovery of new tethering platforms and specific machinery such as FERARI [16] will provide mechanistic insights into the regulation of kiss-and-run events.

## FUSING

The fusion of two membrane compartments is the best studied intracellular communication due its prominent role in the biosynthetic and endocytic pathways. Similar to kiss-and-run, one membrane compartment i.e. vesicle is first tethered, then docks and finally fuses with the acceptor compartment. While in most instances the docked state is relatively short lived, a vesicle can also remain in the docked state for longer periods; for example, in secretory granules at the plasma membrane, where a signal triggers the fusion event and cargo release. In contrast to kiss-and-run, however, the fusion is complete in that the entire membrane of the vesicle is absorbed into the limiting membrane of the acceptor organelle and there is unidirectional cargo exchange. Recently, vesicle transport was also observed with other organelles. Mitochondria are able to generate transport vesicles that are targeted to and fuse with late endosomes or peroxisomes [21, 22]. Likewise, TGN-derived vesicles can fuse with mitochondria at ER-mitochondria contact sites to drive mitochondrial division [23]. Likewise, COPI vesicles fuse with LDs to deliver specific LD proteins [24, 25]. Whether the fusion processes with mitochondria and LD are using the same mechanism as the ‘classical' fusion mechanisms needs to be established. Neither tethers nor SNAREs, which provide specificity in membrane fusion and help to overcome the energy barrier to drive fusion, have been identified. It is conceivable that at least vesicle fusion with the LD follows a distinct mechanism because in this instance a membrane bilayer (vesicle) fuses with a monolayer (LD).

## CONCLUSIONS

Thus touching, kissing and fusing provide a necessary and important repertoire of intracellular organellar communications, the regulation of which we still do not fully understand. Even though a number of protein components for different organellar contact sites have been identified, the regulation of their formation and dynamics and how these processes are linked to efficient lipid and ion transport and to organellar homeostasis remain enigmatic. The interorganellar communication through kiss-and-run is still in its infancy. Until recently we were not even able to detect this process due to its high dynamics and the detection was only made possible through technological advances. This type of communication will increase the versatility and robustness of intracellular transport and presumably aids the cell in ever changing environments to quickly adjust to stress and insults due its potential adaptability. Yet, we will first need to establish how widely this process is used in the cell, which is clearly in the reach given the technological advances in imaging and genome manipulation in metazoans. Yet with these new developments, there is also a need to revisit the process we thought we had a pretty good understanding of: vesicle fusion in intracellular transport and secretion. Clearly the selectivity of the SNARE hypothesis is not sufficient to account for the specificity of all fusion events, and whether SNARE regulators such are SM proteins are enough to provide the additional layer of regulations is debatable. Therefore, the quest of understanding intracellular communication is far from over as new principles and new regulatory circuits are being discovered.

## Abbreviations:

ER: – endoplasmic reticulum,
LD: – lipid droplet,
TGN: – trans-Golgi network.
